# Liquid/solution-based microfluidic quantum dots light-emitting diodes for high-colour-purity light emission

**DOI:** 10.1038/s41598-020-70838-w

**Published:** 2020-09-03

**Authors:** Masahiro Kawamura, Hiroyuki Kuwae, Takumi Kamibayashi, Juro Oshima, Takashi Kasahara, Shuichi Shoji, Jun Mizuno

**Affiliations:** 1 Department of Electronic and Physical Systems, Waseda University, 3-4-1 Okubo, Shinjuku, Tokyo, 169-8555 Japan; 2 Research Organization for Nano and Life Innovation, Waseda University, 513 Waseda Tsurumaki, Shinjuku, Tokyo, 162-0041 Japan; 3 Frontier Materials Research Department, Materials Research Laboratories, Nissan Chemical Corporation, Suzumi, Funabashi, Chiba 274-0052 Japan; 4 Department of Electrical and Electronic Engineering, Faculty of Science and Engineering, Hosei University, Koganei, Tokyo, 184-8584 Japan; 5 Organization for Regional Collaborative Research and Development, Tokyo University of Science, Suwa, Toyohira, Chino, Nagano 391-0292 Japan

**Keywords:** Lasers, LEDs and light sources, Optical materials and structures, Optofluidics

## Abstract

Organic light-emitting diodes (OLEDs) using a liquid organic semiconductor (LOS) are expected to provide extremely flexible displays. Recently, microfluidic OLEDs were developed to integrate and control a LOS in a device combined with microfluidic technology. However, LOS-based OLEDs show poor-colour-purity light emissions owing to their wide full width at half maximum (FWHM). Here we report liquid/solution-based microfluidic quantum dots light-emitting diodes (QLEDs) for high-colour-purity light emission. Microfluidic QLEDs contain liquid materials of LOS for a backlight and QDs solutions as luminophores. The microfluidic QLED exhibits red, green, and blue light emissions and achieves the highest light colour purity ever reported among LOS-based devices for green and red lights with narrow FWHMs of 26.2 nm and 25.0 nm, respectively. Additionally, the effect of the channel depth for the luminophore on the peak wavelength and FWHM is revealed. The developed device extends the capabilities of flexible microfluidic OLEDs-based and QDs-based displays.

## Introduction

Organic light-emitting diodes (OLEDs), composed of organic semiconductors, have been applied in various devices as displays owning to their superior features than liquid crystal displays such as wide view angle, light weight, flexibility, and short response time^1–3^. Recently, solvent-free organic liquids were reported^4–7^. These are π-conjugated moieties with bulky and flexible side chains, which lower the melting point of the molecules, so they adopt a liquid state without any solvents at room temperature. Although characteristics of devices using such liquid materials are inferior than that of devices using solid materials, the use of liquid materials prevents detachment between the liquid layer and electrodes even if device shape changes. In addition, conventional solid type-organic semiconductors are more flexible compared with inorganic counterparts. However, they show poor resistance under large deformation such as stretching and bending due to their inherent rigidity and highly ordered structures^8^. On the other hand, solvent-free organic liquids have not any limitation in flexibility because they are fluidic materials. Consequently, the solvent-free organic liquids are expected to realize truly flexible devices. D. Xu and C. Adachi developed liquid OLEDs using a solvent-free organic liquid named a liquid organic semiconductor (LOS) as an emitting layer^9^. An LOS shows strong charge transfer and fluidity in the neat state^10^. Moreover, liquid OLEDs are able to overcome the short lifetime of solid OLEDs by replacing the degraded LOS with a fresh sample^9,11^. In 2013, we applied microfluidic technology to the liquid OLEDs to integrate the LOS on a chip, and coined the term microfluidic OLEDs^12–16^. Microfluidic OLEDs show various functionalities using the liquid features of LOS such as high flexibility^13^, on-demand colour tunability^14^, and emission recoverability^14^. However, liquid OLEDs show a wide full width at half maximum (FWHM) of 70–100 nm, as do conventional solid OLEDs^11,14,17–19^, owing to structural relaxation in the excited states^19^ and molecular vibration^20^. Such a wide FWHM leads to poor-colour-purity light that does not meet the requirements for 4 K/8 K ultra high definition displays.

However, quantum dots (QDs) are widely studied for high-colour-purity displays. QDs show superior photophysical properties such as narrow FWHM (< 30 nm) emission, broad absorbance band, facile colour tuning in the visible light region, and high photoluminescence (PL) quantum yield^21–23^. Based on these advantages, quantum dots light-emitting diodes (QLEDs), which contain a QDs light-emitting layer, can realize narrow FWHM emissions. QLEDs can be separated into two categories; the first category uses QDs as electroluminescent materials excited by direct carrier injection^24,25^, and the other category uses QDs as colour converters excited by a blue OLED backlight^26–28^. Interestingly, QLEDs with an excitation backlight have been common components in high-colour-purity display applications. The advantage of the high PL quantum yield of QDs leads to a high efficiency of the QLEDs. In addition, the QDs not only work as colour converters, but also as colour filters to cut-off the excess backlight owing to their broad absorbance^29,30^.

**Figure 1:**
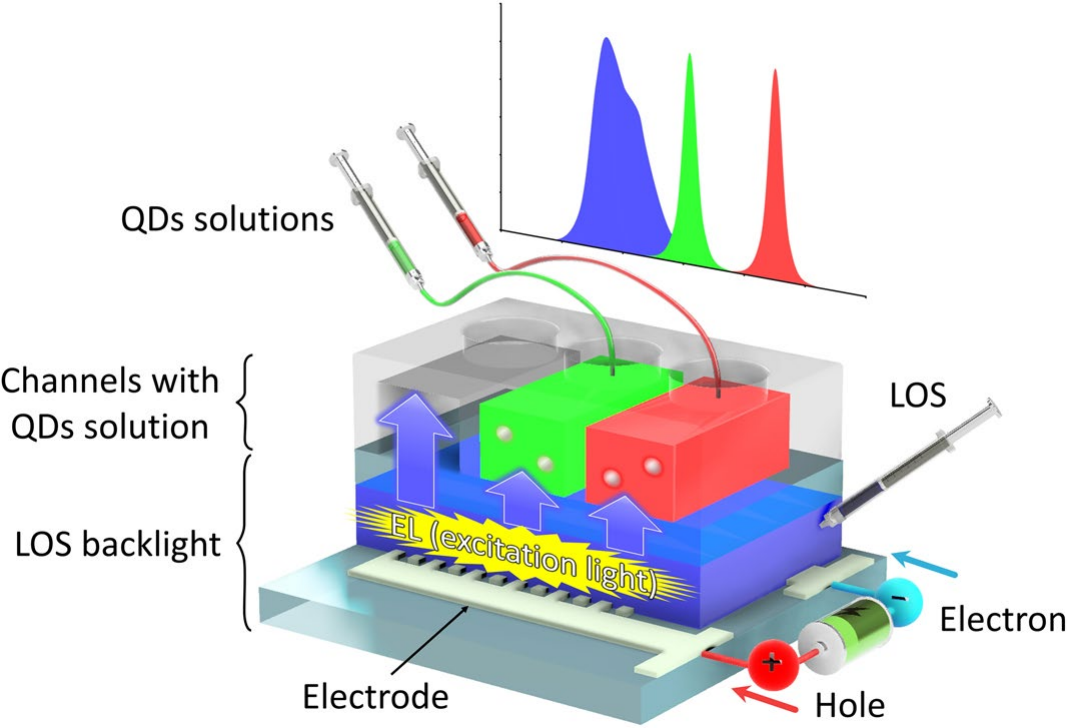
Concept of the liquid/solution-based microfluidic QLEDs. Proposed microfluidic QLEDs consist of a LOS backlight and a QDs solutions layer. The channels for the QDs solutions are stacked on the LOS backlight. The QDs solutions work as colour converters and colour filters to emit high-colour-purity lights with narrow FWHMs.

In this study, we developed liquid/solution-based microfluidic QLEDs, composed of liquid OLEDs as a LOS backlight and QDs solution luminophores, for narrow FWHM emission. The all emission layers, backlight and QDs emission layer, consist of liquid materials. Figure 1 shows the concept of the microfluidic QLEDs. Microchannels filled with water solutions of the QDs were stacked on the LOS backlight. The liquid light-emitting layers were formed by capillary action without any vacuum process^11^. The electroluminescence (EL) of the LOS backlight was generated by applying a voltage. Subsequently, the energy of the light was transferred to the QDs solutions and the QDs emitted high-colour-purity PL with a narrow FWHM. In addition, the excess EL from the LOS backlight was blocked by the filtering effect of the QDs solutions. The microfluidic QLEDs achieved red, green, and blue (RGB) light emissions. Moreover, the relationships between the depth of the channels for the QDs solutions and the FWHM as well as the peak wavelength of the QDs solutions were revealed. Especially, the effect of thickness of thickness of QDs layer on FWHM have not been reported in previous work. The proposed microfluidic QLEDs are expected to exhibit the advantages of microfluidic OLEDs such as high flexibility, on-demand colour tuning, and emission recovery. Some of the content in this paper has been reported in the proceedings of the 20th International Conference on Solid-State Sensors, Actuators and Microsystems (Transducers 2019)^31^, and the proceedings of the 33rd International Conference on Micro Electro Mechanical Systems (MEMS 2020)^32^. Electrical operation results of the LOS backlight, emission image of microfluidic QLED, and relationship between spectral properties and channel depth of the microfluidic QLED are newly reported in this manuscript. The scientific discussion about the all results are also added.

## Results and discussion

### Optical properties of the materials

**Figure 2:**
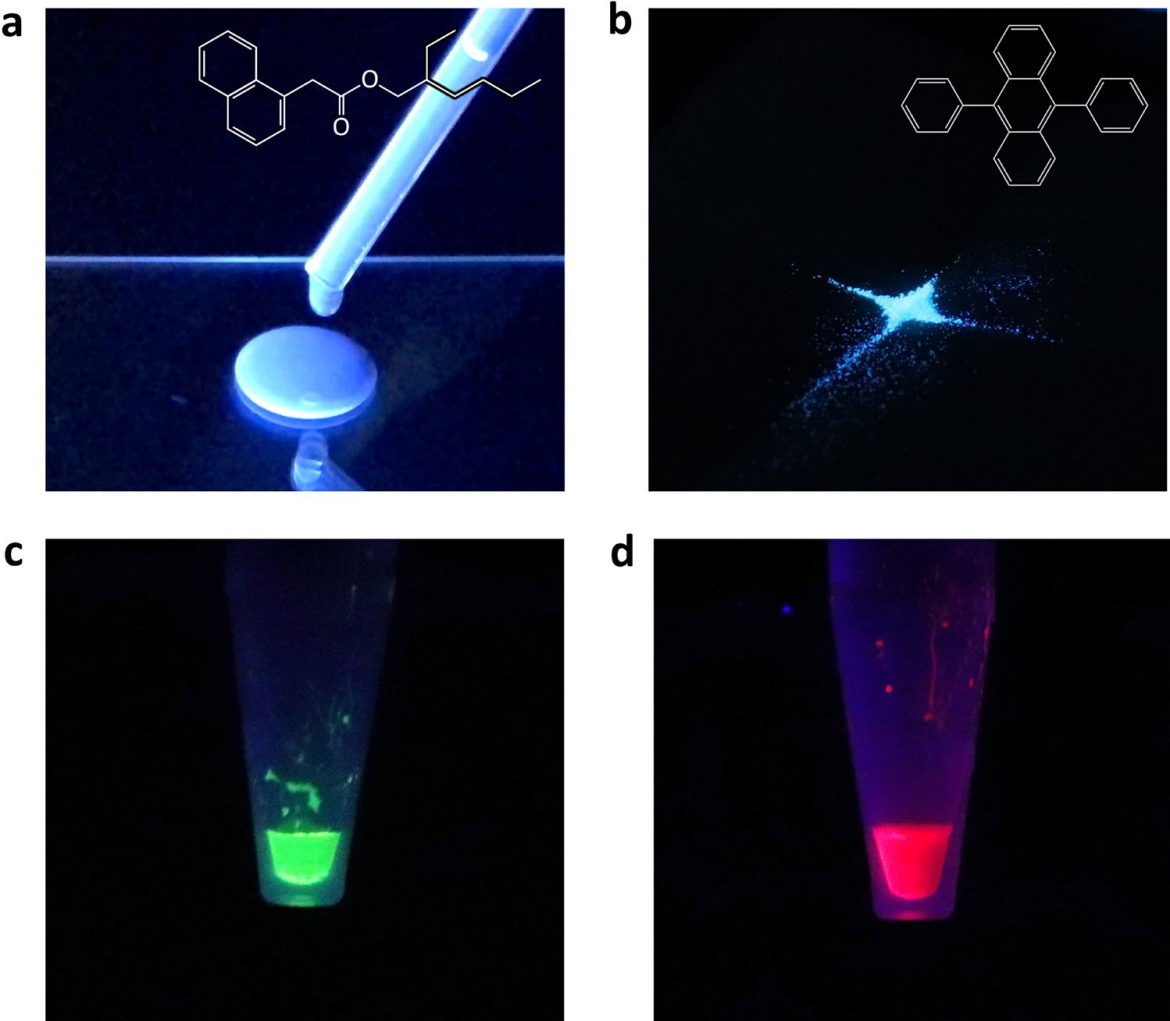
PL images of the LOS and luminophore materials. (**a)** PL image and chemical structure of NLQ, which was used as a host molecule for the liquid light-emitting layer of the LOS backlight. (**b**) PL image and chemical structure of DPA, which was doped in NLQ as a guest molecule to obtain deep-blue EL emission. (**c**,**d**) PL images of green (**c**) and red (**d**) QDs water solutions. All materials were excited by 365-nm UV light.

**Figure 3:**
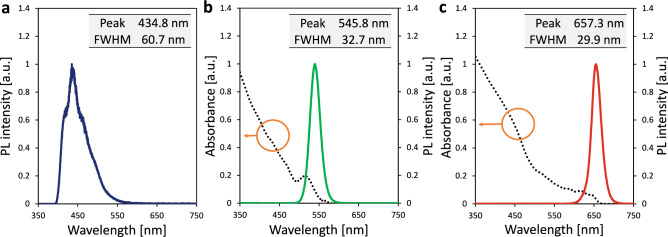
Optical properties of the LOS and luminophore materials. (**a**) PL spectrum of DPA-doped NLQ for the deep-blue LOS backlight. The inset table show the peak wavelength and FWHM of the PL spectrum. (**b**,**c**) PL and absorption spectra of green (**b**) and red (**c**) QDs water solutions. The green and red lines represent PL spectra of each QDs water solution. The black dotted lines represent absorption spectra. The inset tables show the peak wavelengths and FWHMs of the PL spectra.

PL images of the materials of the LOS backlight and QDs solution luminophore are depicted in Fig. 2. 1-naphthaleneacetic acid 2-ethylhexyl ester (NLQ) (Fig. 2a), which has the widest HOMO–LUMO gap (4.08 eV) for a LOS ever reported, and 9,10-diphenylanthracene (DPA) (Fig. 2b), which is a dopant already used in solid-OLEDs to produce deep-blue light, were employed as host and guest molecules, respectively. This host–guest system of liquid-OLEDs, reported in our previous research^18^, shows the deepest-blue light emission ever reported. 2.0 wt% of DPA was doped into NLQ, and 0.25 wt% of tributylmethylphosphonium bis(trifluoromethanesulfonyl) imide (TMP-TFSI), as an electrolyte, was also introduced into NLQ to improve the efficiency of carrier injection^11,12^. 0.8 µM carboxyl functionalized CdSeS/ZnS core–shell type QDs water solutions worked as green and red luminophores (Fig. 2c,d) to yield a narrow FWHM for high-colour-purity emission. Optical properties of DPA doped NLQ, which is a liquid light-emitting layer, and the green and red QDs water solutions are shown in Fig. 3. The PL spectra of each of the materials were measured under 365-nm ultraviolet (UV) irradiation. The absorbances of the QDs water solutions were measured at a concentration of 0.08 µM to prevent reaching the measurement limit of the system by UV–visible spectroscopy. As shown in Fig. 3a, the liquid light-emitting layer emitted deep-blue PL at 434.8 nm with a wide FWHM of 60.7 nm. The component of neat NLQ, which has a peak wavelength at 395 nm^17^, was not observed. This result suggested that Förster resonance energy transfer occurred from the host molecule of NLQ to the guest molecule of DPA because of the overlap between the PL spectrum of the NLQ host and the absorption spectrum of DPA^18^. Furthermore, green and red QDs showed green and red PL emissions at 545.8 and 655.1 nm with narrow FWHMs of 32.7 and 28.6 nm, respectively (Fig. 3b,c). The green and red QDs absorbed the light with a shorter wavelength than 574 and 664 nm, respectively, and these absorptions had a large overlap with the PL spectra of DPA-doped NLQ, which was employed as the backlight. The DPA-doped NLQ based backlight was suitable as an excitation light source for the green and red QDs water solutions, and the proposed concept, a liquid/solution-based microfluidic QLED, was expected to realize high-colour-purity and narrow FWHM light emission.

### Electrical operation of the LOS backlight

**Figure 4:**
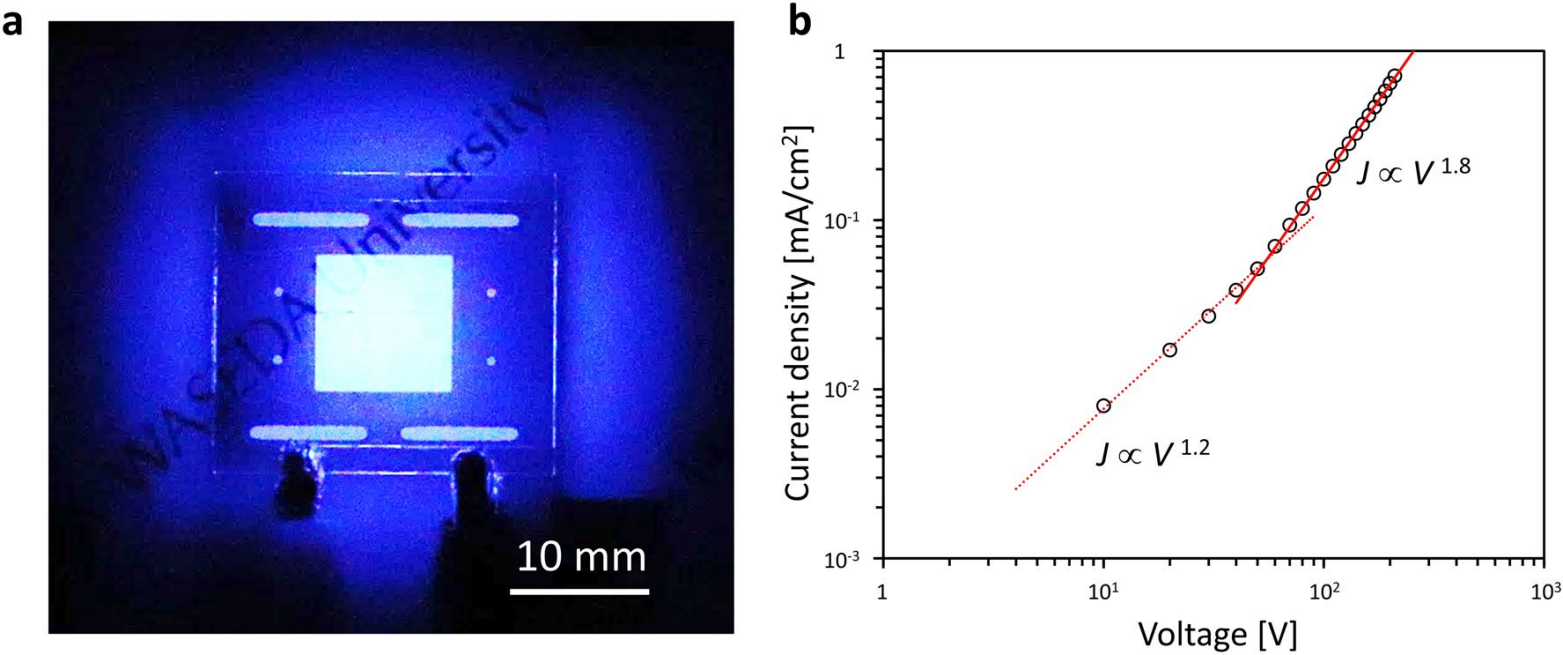
Electrical operation results of the LOS backlight. (**a**) EL image of the LOS backlight with an applied voltage of 200 V. (**b**) *J–V* curve of the LOS backlight. The red dotted line represents a power approximation of the data below 50 V. The red solid line represents a power approximation of the data above 50 V.

The LOS backlight was prepared in a simple structure containing the liquid light-emitting layer on a comb indium tin oxide (ITO) electrode (Supplementary Figure S1 (a)). As shown in Fig. 4a, the deep-blue EL light was successfully observed by applying a voltage. The EL spectrum of the LOS backlight had a peak wavelength of 436.5 nm, which is almost the same as that of the PL spectrum of DPA-doped NLQ (434.8 nm). However, the FWHM of the EL light emission was 10.4 nm wider than that of the PL light emission. Such a FWHM broadening was attributed to Joule heating owing to the carrier flow in the liquid light-emitting layer, which had poor thermal and electrical conductivity^33,34^. The current density–voltage (*J*–*V*) curve corresponded to the ohmic law below 50 V and the space charge limited current law above 50 V (Fig. 4b). This was the same behaviour as that for the previously reported liquid OLEDs^11,12^. These results indicated that the operation of the DPA-doped NLQ based LOS backlight was successful with a stable carrier injection from ITO electrodes to the liquid light-emitting layer. This is the first report of operating liquid OLEDs with a comb electrode.

### Electrical operation of the microfluidic QLED

**Figure 5:**
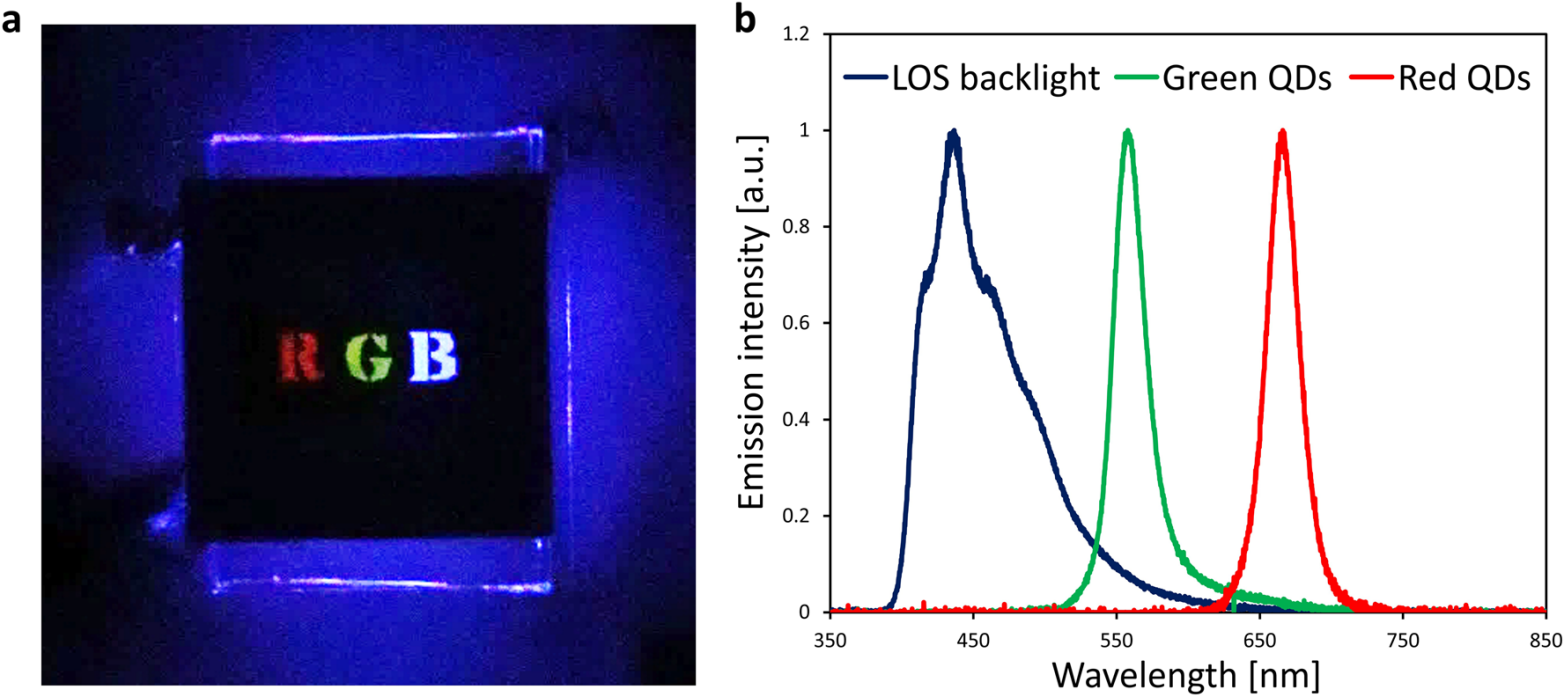
Electrical operation results of the microfluidic QLED. (**a**) Electrical operation image of the microfluidic QLED with an applied voltage of 200 V. A patterned mask was stacked on the PDMS channels to depict RGB. (**b**) Emission spectra of the microfluidic QLED. Each spectrum was measured with an applied voltage of 200 V. The inset table shows the peak wavelengths and FWHMs of each spectrum.

The structure of the liquid/solution-based microfluidic QLEDs is shown in Supplementary Figure S1 (b). The luminophore channels, which were filled with water solutions of the QDs, were prepared using a poly(dimethylsiloxane) (PDMS) film by soft lithography, and then stacked on the LOS backlight. We applied 200 V to the LOS backlight for the stable operation, with consideration of the *J–V* curve of the LOS backlight. RGB lights with green and red PL emissions of the QDs water solutions and the deep-blue EL emission of the LOS backlight were successfully observed (Fig. 5a). A patterned mask was stacked on the PDMS channels to depict RGB. As shown in Fig. 5b, the EL emission of the LOS backlight was not observed in the emission spectra of the green and red lights because of the strong colour filtering effect of the QDs solutions. Table 1 shows peak wavelengths and FWHMs of PL spectra excited by 365-nm UV light and electrical operation results with the LOS backlight. The peak wavelengths were 557.3 nm and 665.6 nm for the emissions of the green and red lights. Moreover, the green and red lights from the QDs water solutions had narrow FWHMs of 26.2 and 25.0 nm, respectively. The PL emission was generated by energy transfer from the higher energy light of the LOS backlight. Compared with the PL spectra of each of the QDs water solutions, a red-shift of the peak wavelength and FWHM narrowing were observed in the emission spectra of the microfluidic QLED.

**Table 1:** Summary of the light emission spectral properties.

Colour	PL excitation with 365-nm UV light		Electrical operation with the LOS backlight	
	Peak (nm)	FWHM (nm)	Peak (nm)	FWHM (nm)
Blue	434.8	60.7	436.5	71.0
Green	545.8	32.7	557.3	26.2
Red	657.3	29.9	665.6	25.0

**Figure 6:**
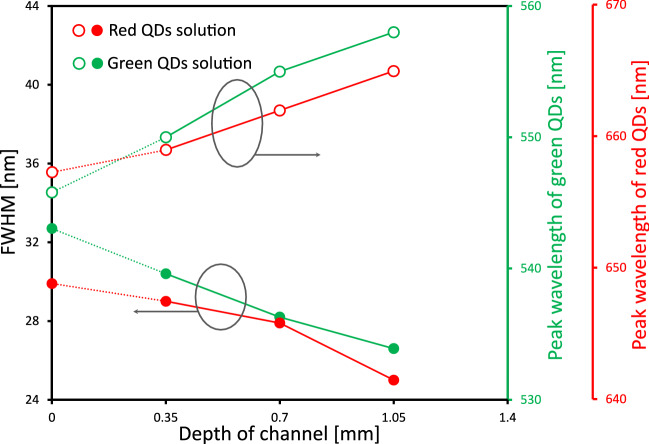
Effect of channel depth on emission spectra of the microfluidic QLED. Relationship between the channel depth and emission spectra properties such as peak wavelengths and FWHMs. The filled and open circles represent FWHMs and peak wavelengths, respectively. The data for depth of channel = 0 mm were measured by a detection probe at the same point as the excitation with 365-nm UV light. The other data were estimated from emission spectra of the microfluidic QLED with different channel depths (see Supplementary Figure S2).

Here, we discuss the mechanism of the red-shift and FWHM narrowing of the QDs solutions. The emission spectra of the microfluidic QLEDs with different depths of the channels for the QDs luminophores excited by the LOS backlight were determined (see the supplementary information for the details of the experiment and raw data of the emission spectra (Supplementary Figure S2). The data for depth = 0 mm were measured by a detection probe at the same point as the excitation with 365-nm UV light. As shown in Fig. 6, the red-shift and FWHM narrowing of the PL spectra were accelerated by increasing the depth of the channels for the green and red QDs water solutions. The fluorescence quenching due to high concentration of QDs particles is occurred more than 2 µM^35^, although the concentration of the QDs solutions used in the devices was 0.8 µM. Thus, the effect of concentration quenching of QDs solutions could be negligible. The main cause of these phenomena was effect of reabsorption of the QDs due to the size distribution of the particles^36,37^. QDs particles show size dependent bandgap due to quantum confinement effects. The large QDs particles absorb the lights with longer wavelength than the small ones because the larger QDs particles have narrower energy bandgap. Energy transfer from the excited small QDs particles to the large QDs particles occurred owing to the reabsorption of the larger QDs particles. The light emission of the large QDs became the dominant emission wavelength component because of the energy transfer between the small and large QDs, which led to the red-shift of the emission. Moreover, the energy transfer also suppressed the spectral distribution of the PL emission of the QDs due to the reabsorption caused by the size distribution of the QDs particles, which led to FWHM narrowing of the emission. These phenomena strongly appeared as the depth of the channel increased. In this study, the excitation and detection points were the same while measuring the PL spectra of the QDs solutions. However, when the microfluidic QLED was operated, the excitation light of the LOS backlight and the emission light were detected by a probe which was placed above the channels. This difference caused the red-shift and FWHM narrowing of the emission (Supplementary Figure S3). From these results, it was revealed that the peak wavelength and FWHM of the emission from a microfluidic QLED could be controlled by adjusting the depth of the channel for the QDs luminophores.

**Figure 7:**
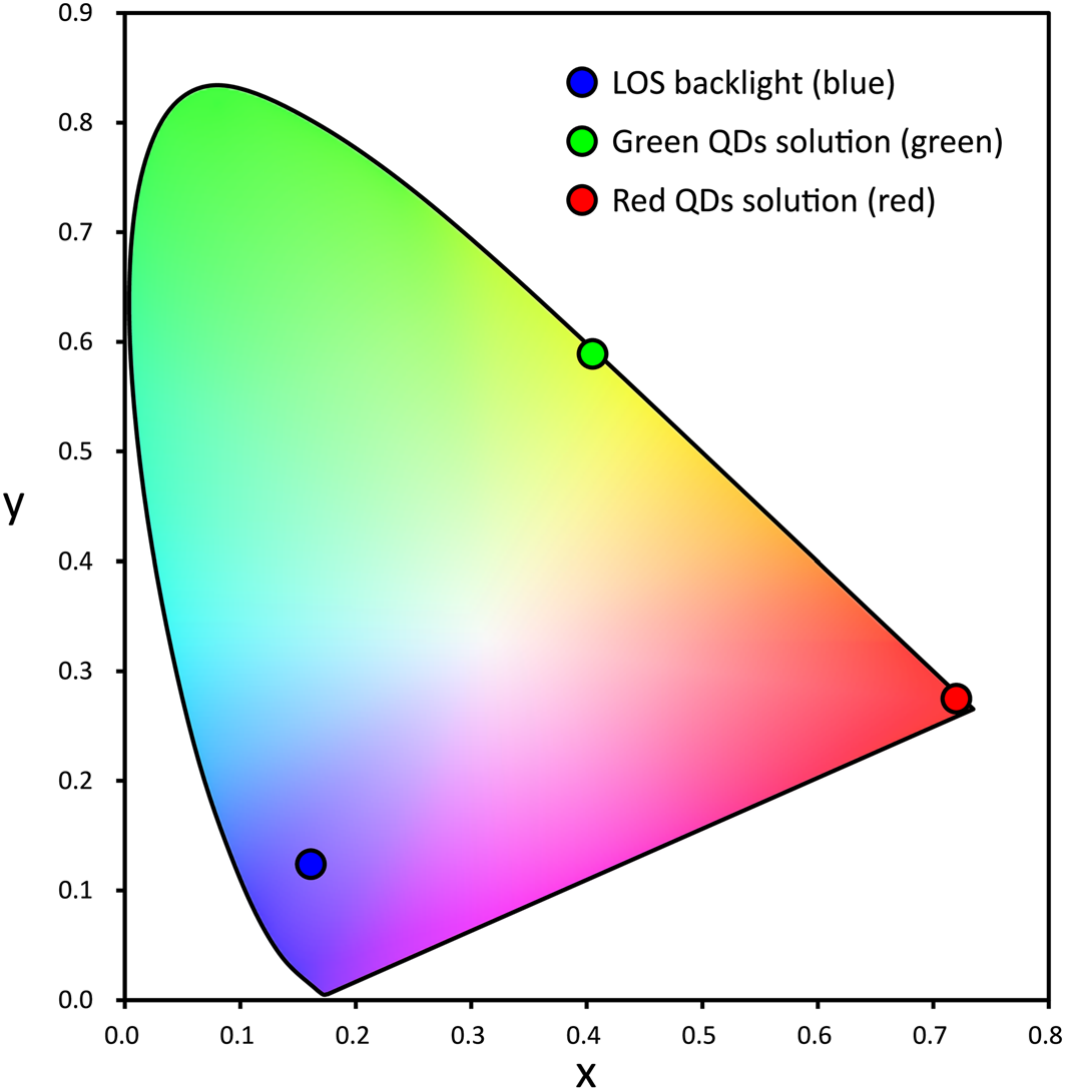
CIE 1931 RGB colour space of the lights emitted from the microfluidic QLEDs. The CIE coordinates of the RGB lights were calculated from the emission spectra (Fig. 5b) using the software colorAC. The coordinates of (0.33, 0.33) is the lowest colour purity point in the CIE colour space. The light colour purity become higher as the plot get closer to the outside perimeter.

The colour purities of the obtained light emissions were evaluated by Commission Internationale de l’Eclairage (CIE) 1931 RGB colour space, as shown in Fig. 7. The CIE coordinates of the blue, green, and red lights emitted from the microfluidic QLED were calculated from the emission spectra using the CIE coordinate calculator software ColorAC. The CIE coordinates of the emission from the blue, green, and red lights were (0.161, 0.124), (0.405, 0.589), and (0.720, 0.275), respectively. In the CIE colour space, as a plot gets closer to the outer periphery, known as the spectrum locus and purple boundary, it means a higher colour purity. The green and red lights of the QDs water solutions had a high colour purity owing to their narrow FWHMs after converting light energy from the blue LOS backlight with a wide FWHM. In addition, the green light was the practically greenest light with high-colour-purity in the device since a QDs solution with greener light (near the edge of the CIE colour space) would be unable to filter the EL light of the LOS backlight completely (details of the consideration are shown in Supplementary Figure S4 and S5). The colour purity of lights from the microfluidic QLED is the highest in LOS-based devices ever reported^9,14,17^.

## Conclusion

We proposed liquid/solution-based microfluidic QLEDs using a deep-blue LOS backlight and QDs water solutions to realize high colour purity light emission. A QDs luminophore layer was prepared using PDMS films containing channels filled with QDs water solutions. The LOS backlight using DPA-doped NLQ showed a deep-blue EL emission. RGB light emissions from the device were successfully observed by applying a voltage. The QDs water solutions excited by the LOS backlight emitted vibrant green and red PL light with narrow FWHMs of 26.2 nm and 25.0 nm, respectively. As the depth of the channels for the QDs water solutions increased, the PL emission of the QDs water solutions showed a strong red-shift and FWHM narrowing owing to the size dispersion of QDs particles and the points of excitation and detection. In the CIE 1931 colour space, the coordinates of green and red PL lights were on the boundary, which means ultra-high-colour-purity light. The colour purities were the highest of the LOS-based devices reported to date. Therefore, the liquid/solution-based microfluidic QLEDs are a promising step for next-generation high-colour-purity flexible displays. However, high-colour purity of the emission from blue and true green lights has not been realized yet owing to a large cross talk of the emission spectra of the LOS backlight and the blue- and true green-QDs water solutions. We expect that development of deeper blue LOS backlight, can realized further high-colour-purity LOS based QLEDs devices.

## Methods

### Materials

1-naphthaleneacetic acid 2-ethylhexyl ester was supplied by Nissan Chemical Corporation, Ltd. (Tokyo, Japan). 9,10-diphenylanthracene and tributylmethylphosphonium bis(trifluoromethanesulfonyl) imide were purchased from Tokyo Chemical Industry Co., Ltd. (Tokyo, Japan). To fabricate the liquid light-emitting layer, NLQ, DPA, and TMP-TFSI were dissolved and mixed into dichloromethane. Then, the solution was put in a vacuum oven at 80 ℃ for 3 h to evaporate the solvent completely. The concentration of DPA and TMP-TFSI against NLQ was 2 wt% and 0.25 wt%, respectively. The red carboxyl-functionalized CdSeS/ZnS core–shell type QDs water solution was purchased from Thermo Fisher Scientific Co., Ltd. (Tokyo, Japan). The green-type QDs water solutions were purchased from Sigma-Aldrich Co., Ltd. (Tokyo, Japan). The shell which coats the core improve the quantum yield of QDs by passivizing nonradiative recombination sites. The red and green QDs solutions show high PL quantum yields of more than 50%. 0.8 µM QDs solutions were used in the microfluidic QLEDs and PL measurements. 0.08 µM QDs solutions were used for measuring their absorption spectra. The PL quantum yields are maintained when they dilute and disperse completely^38^.

### Device fabrication

Laminated silicon chips (2 mm × 20 mm × 1.05 mm) were used as a mould for the channel fabrication. PDMS was cast over the mould, then annealed at 100 °C for 30 min for curing. After releasing the PDMS channels, inlets and outlets were formed. The LOS backlight was fabricated using a cell with a 10-μm-pitch comb electrode (EHC Co., Ltd.). Subsequently, the PDMS channels were stacked on the LOS backlight. Finally, DPA doped NLQ with TMP-TFSI and QDs solutions were injected into the device. The solutions of the QDs might cause instability due to the solution evaporation. It could be solved by employing non-volatile QDs liquids, which shows liquid state at room temperature without any solvents thanks to ligands around QDs particles, instead of QDs solutions^39–41^.

## Data availability

The relevant data are available from the authors upon reasonable request.

## Supplementary information

Supplementary information.
