# Supplementary material for: Liquid/solution-based microfluidic quantum dots light-emitting diodes for high-colour-purity light emission
Source: Sci Rep. 2020 Sep 3;10:14528. doi: 10.1038/s41598-020-70838-w (PMC7471114; doi:10.1038/s41598-020-70838-w)
Supplement: Supplementary file 1 — Supplementary information. [file 41598_2020_70838_MOESM1_ESM.pdf]

Liquid/solution-based microfluidic quantum dots light-emitting diodes for high-colour-purity light emission

Masahiro Kawamura[1], Hiroyuki Kuwae[1,2,*], Takumi Kamibayashi[1], Juro Oshima[3], Takashi Kasahara[4], Shuichi Shoji[1], and Jun Mizuno[2,5]

[1]Department of Electronic and Physical Systems, Waseda University, 3-4-1 Okubo, Shinjuku, Tokyo 169-8555, Japan.

[2]Research Organization for Nano and Life Innovation, Waseda University, 513 Waseda Tsurumaki, Shinjuku, Tokyo 162-0041, Japan.

[3]Frontier Materials Research Department, Materials Research Laboratories, Nissan Chemical Corporation, 488-6 Suzumi, Funabashi, Chiba 274-0052, Japan.

[4]Department of Electrical and Electronic Engineering, Faculty of Science and Engineering, Hosei University, Koganei, Tokyo 184-8584, Japan.

[5]Organization for Regional Collaborative Research and Development, Tokyo University of Science, Suwa, Toyohira, Chino, Nagano 391-0292, Japan.

[*] Author to whom correspondence should be addressed. kuwae@shoji.comm.waseda.ac.jp

This Word document file includes:
Supplementary text
Supplementary Figures S1, S2, S3, S4, and S5

List of Supplementary Figures

Supplementary Figure S1 | Experimental device setup for (a) the LOS backlight and (b) the microfluidic QLED.

Supplementary Figure S2 | Emission spectra of the (a) red and (b) green microfluidic QLED with different depths of the channel of 0.35 mm, 0.70 mm, and 1.05 mm.

Supplementary Figure S3 | Experimental setup used to evaluate the relationship between the excitation position and the detection position. The detection position was (a) the same side as the excitation light, and (b) the opposite side as the excitation light across the channel. (c) Emission spectra measured in the experimental setup of (a) and (b). Inset: (c) peak wavelength and FWHM of the PL spectra.

Supplementary Figure S4 | Emission spectra of microfluidic QLED using green-type QDs solutions with a peak wavelength of (a) 495.2 nm, (b) 523.5 nm, and (c) 545.8 nm. Inset: peak wavelength and FWHM of each spectrum.

Supplementary Figure S5 | Summary of CIE 1931 RGB colour space of the all electrical operation results in this research.

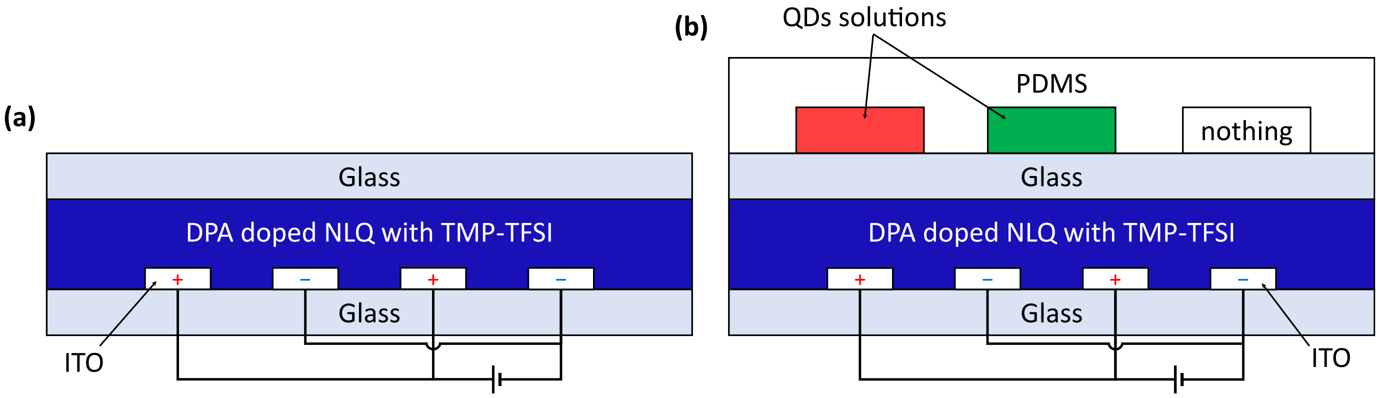

Figure S1. | Experimental device setup for (a) the LOS backlight and (b) the microfluidic QLED. Supplementary Figure S1 shows the cross-sectional view of the LOS backlight and the microfluidic QLED used in the experiment.

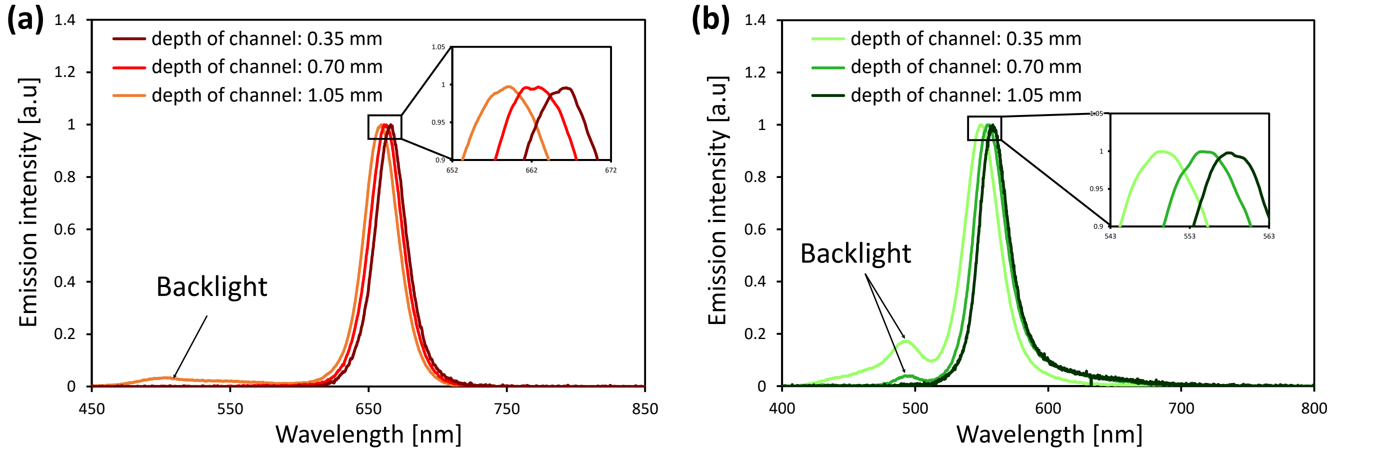

Figure S2. | Emission spectra of the (a) red and (b) green microfluidic QLED with different depths of the channel of 0.35 mm, 0.70 mm, and 1.05 mm. Supplementary Figure S2 shows the changes of the emission spectra for the microfluidic QLEDs with an increasing depth of the channel for the QDs solutions luminophore. The remaining EL spectrum of the LOS backlight was observed around 500 nm in the spectra with a channel depth of 0.35 mm. However, the EL spectrum was suppressed with increasing the channel depth. In the red-microfluidic QLED with a channel depth deeper than 0.70 mm, all the remaining EL light from the LOS backlight was absorbed and filtered by the QDs solution. However, a channel depth of 1.05 mm was required in the green-microfluidic QLED for the remaining EL light to disappear. Moreover, the peak wavelength showed a red-shift and the FWHM became narrower as the depth of the channel increased.

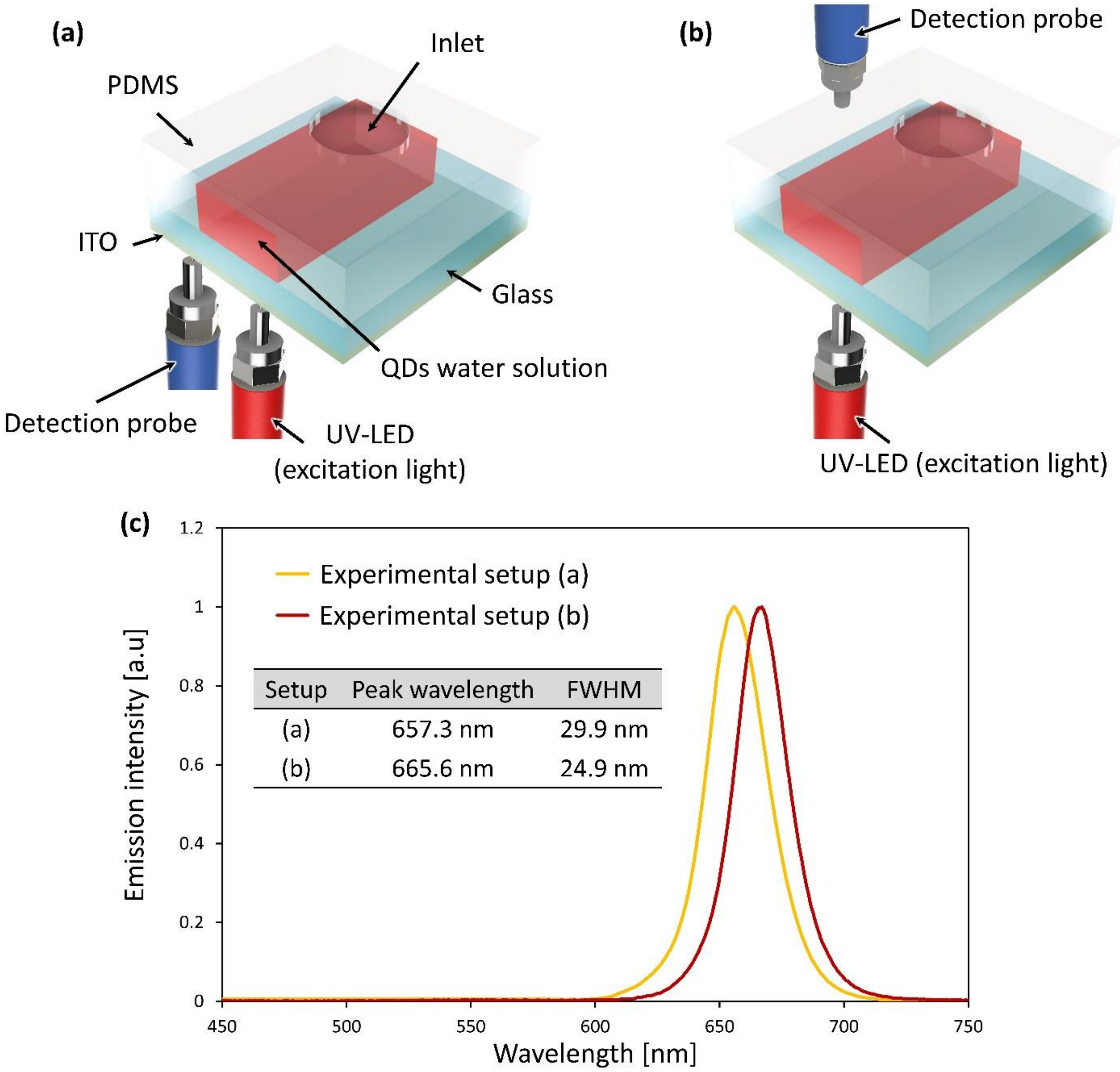

Figure S3. | Experimental setup used to evaluate the relationship between the excitation position and the detection position. The detection position was (a) the same side as the excitation light, and (b) the opposite side as the excitation light across the channel. (c) Emission spectra measured in the experimental setup of (a) and (b). Inset: (c) peak wavelength and FWHM of the PL spectra. Supplementary Figure S3 shows the emission spectral changes with changing the excitation and detection positions. The PDMS channel filled with the red QDs water solution was prepared on a ITO/glass substrate. The channel depth was 1.05 nm. The PL spectra were measured using excitation with 365-nm UV light. Compared with the PL spectrum measured in the setup (a), the PL spectrum measured in the setup (b) was red-shifted by 8.3 nm and the FWHM was narrowed by 5 nm. Moreover, the PL spectrum measured in the setup (b) correspond well with the emission spectra from the microfluidic QLEDs (Figure 5b and Table 1).

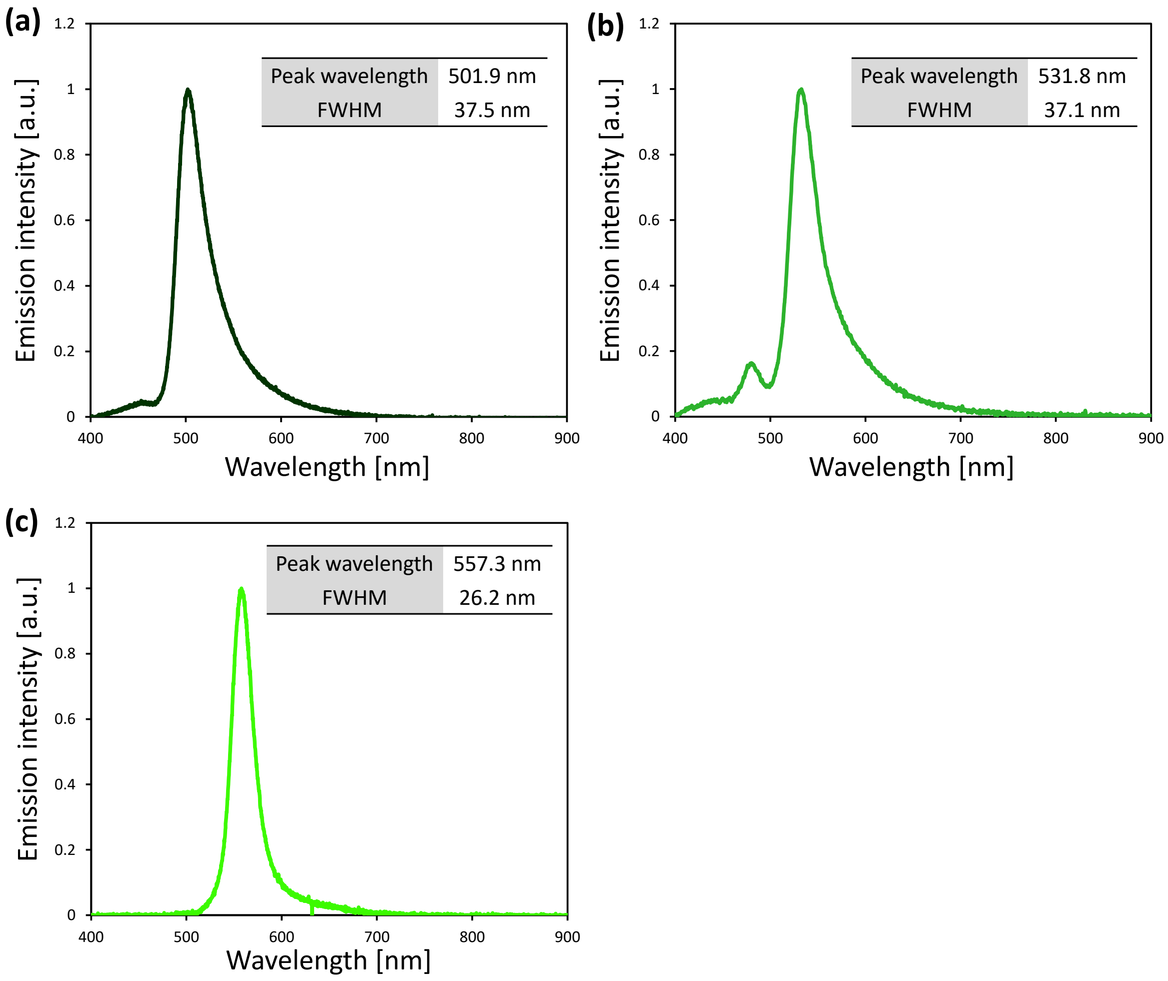

Figure S4. | Emission spectra of microfluidic QLED using green-type QDs solutions with a peak wavelength of (a) 495.2 nm, (b) 523.5 nm, and (c) 545.8 nm at excitation results under 365-nm UV irradiation. Inset: peak wavelength and FWHM of each spectrum. Supplementary Figure S4 shows emission spectra of microfluidic QLEDs using green-type QDs solutions. The concentration of each QDs solution were as same as that of using in the QLEDs device. In addition the green QDs solution (545.8 nm) described in the manuscript, greener QDs solutions with peak wavelength of 495.2 nm and 523.5 nm at excitation results under 365-nm UV irradiation were employed to realize true green light emission. The red-shift of the peak wavelength between excitation results and the QLEDs device (Figure S4) was caused by the differences of the light detection side as discussed in Figure S3. As shown in Supplementary Figure S3(a) and (b), a greener-QDs solution than the green-QDs solution reported in the manuscript (Supplementary Figure S3(c)) can be realized, although the EL light of the LOS backlight strongly affected the emission spectra. This means that the FWHMs of the emission spectra widened compared with that of the original PL spectra of each QDs solution. Thus, the QDs solution used in Supplementary Figure S3(c) showed a practically optimal emission spectrum to realize both the narrowest FWHM and the greenest emission.

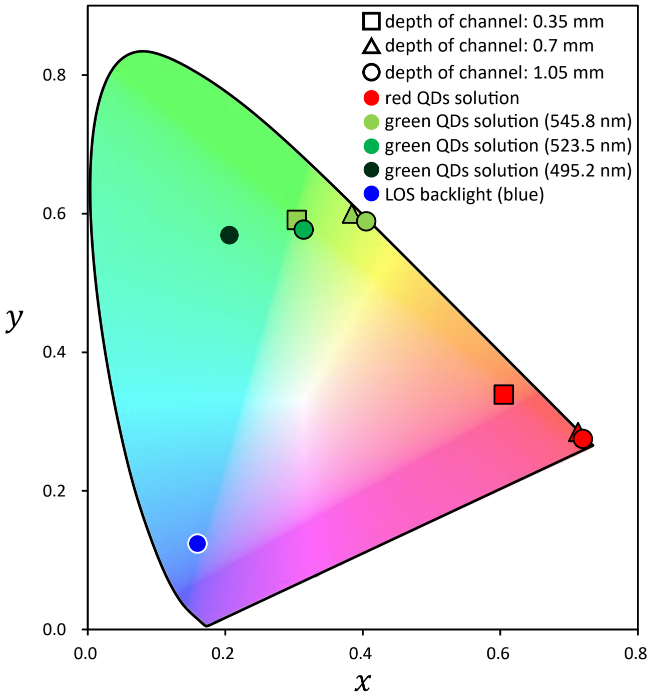

Supplementary Figure S5 | Summary of CIE 1931 RGB colour space of the all electrical operation results in this research. Supplementary Figure S5 shows the CIE coordinates of the light emission from the microfluidic QLEDs for evaluation of light colour purity. The CIE coordinates were calculated from the emission spectra shown Supplementary Figure S2 and S4. As shown in the Figure S5, colour purities of the lights described in the manuscript (Figure 7) are the practically highest.
